# Receptor–Receptor Interactions of G Protein-Coupled Receptors in the Carotid Body: A Working Hypothesis

**DOI:** 10.3389/fphys.2018.00697

**Published:** 2018-06-07

**Authors:** Andrea Porzionato, Elena Stocco, Diego Guidolin, Luigi Agnati, Veronica Macchi, Raffaele De Caro

**Affiliations:** ^1^Department of Neuroscience, University of Padua, Padua, Italy; ^2^Department of Diagnostic, Clinical Medicine and Public Health, University of Modena and Reggio Emilia, Modena, Italy

**Keywords:** carotid body, GPCRs, heteromers, receptor mosaics, hypoxia, adenosine, dopamine, plasticity

## Abstract

In the carotid body (CB), a wide series of neurotransmitters and neuromodulators have been identified. They are mainly produced and released by type I cells and act on many different ionotropic and metabotropic receptors located in afferent nerve fibers, type I and II cells. Most metabotropic receptors are G protein-coupled receptors (GPCRs). In other transfected or native cells, GPCRs have been demonstrated to establish physical receptor–receptor interactions (RRIs) with formation of homo/hetero-complexes (dimers or receptor mosaics) in a dynamic monomer/oligomer equilibrium. RRIs modulate ligand binding, signaling, and internalization of GPCR protomers and they are considered of relevance for physiology, pharmacology, and pathology of the nervous system. We hypothesize that RRI may also occur in the different structural elements of the CB (type I cells, type II cells, and afferent fibers), with potential implications in chemoreception, neuromodulation, and tissue plasticity. This ‘working hypothesis’ is supported by literature data reporting the contemporary expression, in type I cells, type II cells, or afferent terminals, of GPCRs which are able to physically interact with each other to form homo/hetero-complexes. Functional data about cross-talks in the CB between different neurotransmitters/neuromodulators also support the hypothesis. On the basis of the above findings, the most significant homo/hetero-complexes which could be postulated in the CB include receptors for dopamine, adenosine, ATP, opioids, histamine, serotonin, endothelin, galanin, GABA, cannabinoids, angiotensin, neurotensin, and melatonin. From a methodological point of view, future studies should demonstrate the colocalization in close proximity (less than 10 nm) of the above receptors, through biophysical (i.e., bioluminescence/fluorescence resonance energy transfer, protein-fragment complementation assay, total internal reflection fluorescence microscopy, fluorescence correlation spectroscopy and photoactivated localization microscopy, X-ray crystallography) or biochemical (co-immunoprecipitation, *in situ* proximity ligation assay) methods. Moreover, functional approaches will be able to show if ligand binding to one receptor produces changes in the biochemical characteristics (ligand recognition, decoding, and trafficking processes) of the other(s). Plasticity aspects would be also of interest, as development and environmental stimuli (chronic continuous or intermittent hypoxia) produce changes in the expression of certain receptors which could potentially invest the dynamic monomer/oligomer equilibrium of homo/hetero-complexes and the correlated functional implications.

## Introduction

The carotid body (CB) is a small polymodal peripheral chemoreceptor standing out for its basic role in case of hypoxia, hypercapnia, acidosis, low glucose; in these circumstances, it promotes an adequate respiratory and cardiovascular response as well as other less studied stimuli (Atanasova and Lazarov, 2014; Ortega-Sáenz et al., 2015). Two cell types can be distinguished in the CB, the ‘neuron-like’ chemosensitive type I cells and the ‘glial-like’ supportive type II cells. Type I cells synthetize and release many different neurotransmitters and neuromodulators, such as dopamine, acetylcholine, serotonin, histamine, ATP, glutamate, GABA, and substance P. Neurotransmitters and neuromodulators released by type I cells in great measure exert their activity on the afferent endings of the carotid sinus nerve (Porzionato et al., 2018), conveying the stimulation through the glossopharyngeal nerve and petrosal ganglion. The main excitatory stimuli are considered acetylcholine and ATP, binding to the ionotropic nicotinic and P2X_2_/P2X_3_ receptors, respectively. However, autocrine/paracrine modulation of type I cells is also of particular importance and is mainly performed through a series of metabotropic G protein-coupled receptors (GPCRs), such as A_2A_, D_1/2_, H_1/2/3_, M_1/2_, 5-HT_2A,_ and others (for comprehensive reviews on neurotransmitters in the CB, see Iturriaga and Alcayaga, 2004; Bairam and Carroll, 2005; Nurse, 2005, 2014). Conversely, metabotropic GPCRs are also present in afferent nerve endings and type II cells, where they play a role in neuromodulation of signaling between type I cells and afferent fibers (Tse et al., 2012; Nurse et al., 2018). Many authors have emphasized the absolute peculiarity of the CB, characterized by “a plethora of neurotransmitters and neuromodulators, as well as an even broader spectrum of their receptors” (Nurse, 2014). Apart from the above sensory innervation, the CB also shows sensory innervation from jugular and nodose ganglia, post-ganglionic sympathetic nerve fibers from the superior cervical ganglion, and preganglionic parasympathetic and sympathetic fibers reaching ganglion cells in the CB. Efferent parasympathetic and sympathetic innervation of the CB plays a pivotal role in modulation of blood flow (De Caro et al., 2013).

-1The CB also shows a high level of structural plasticity, undergoing structural and functional changes during development (e.g., Porzionato et al., 2008a,b; De Caro et al., 2013), aging (e.g., Di Giulio et al., 2012; Zara et al., 2013) and as a consequence of environmental stimuli, such as chronic continuous hypoxia, taking place during acclimation to high altitude (e.g., Wang and Bisgard, 2002). In response to chronic hypoxia, type II cells can act in a stem cell-like manner, differentiating into precursors of neural cells that originate mature glomus cells (Pardal et al., 2007; Platero-Luengo et al., 2014).

The GPCR family involves about 800 human receptors that are organized into five subfamilies, namely classes A (the largest group), B, C, frizzled, and adhesion (Foord, 2002; Guidolin et al., 2018). Considering the structure, GPCRs have a similar conformation showing a peculiar seven TM (7TM) α-helical region, an extracellular amino-terminal segment and an intracellular carboxy-terminal tail (Tuteja, 2009). While the domain that is localized in the extracellular portion allows the ligand(s) – receptor interaction, the TM region is subject to a conformational change when the binding with a ligand occurs; this change is then transmitted to the intracellular region of the GPCR responsible for activating the signaling cascade (Schonenbach et al., 2015; Stockert and Devi, 2015).

According to evidences from *in vitro* and *in vivo* studies, GPCR monomers can recognize/decode signals (Bayburt et al., 2007; Whorton et al., 2007; Kuszak et al., 2009; Lohse, 2010; Guidolin et al., 2018); in particular, an intrinsic plasticity characterizes GPCR monomers signaling, in fact GPCR activation can determine the onset of different signal transduction patterns, like the G proteins and/or arrestin pathways (Zidar et al., 2009; Guidolin et al., 2018). Furthermore, it is established that GPCRs can functionally interact by sharing signaling pathways or by mechanisms of transactivation (see Köse, 2017; Guidolin et al., 2018). In the 1980s, however, Agnati et al. (2005) provided evidence suggesting that GPCRs could also establish structural receptor–receptor interactions (RRIs; see Guidolin et al., 2015, 2018 for recent reviews), in which they can associate with themselves (homodimerization) or with other proteins of the same family (heterodimerization). This concept was later confirmed in transfected mammalian cell systems but also in many different native tissues (i.e., central nervous system; mammary gland; liver; cancer tissues) (see Ferré et al., 2014; Guidolin et al., 2015). It is a fact that class C GPCRs form constitutive homomers or heteromers (Kniazeff et al., 2011; Guidolin et al., 2018). An example is represented by the metabotropic receptor for gamma-aminobutyric acid b (GABA_B_), that, in the central nervous system, constitutes the major inhibitory neurotransmitter (Calver et al., 2000; Pontier et al., 2006). The receptor for GABA_B_, which is formed by the subunits GABA_B1_ and GABA_B2_, represents the typical example of constitutive heterodimer (Lohse, 2010). Only GABA_B1_ can bind GABA; however, GABA_B2_, even if alone is not functional, is required as GABA_B1_ as such is considered immature due to the presence of a carboxy-terminal ER retention motif (Galvez et al., 2001; Kamal and Jockers, 2011). Hence, after co-expression, the heterodimerization is the prerequisite to permit the masking of the retention domain and the subsequent translation of the active protein to the plasma-membrane (Terrillon and Bouvier, 2004). The oligomerization process in class A GPCR is a broadly debated topic of interest (see Franco et al., 2016; Guidolin et al., 2018). However, according to the consistent results obtained through multiple approaches, the possibility of class A GPCR complexes is strongly supported (Bouvier and Hébert, 2014; Guidolin et al., 2018). In this respect, studies highlighting the occurrence of a specific and dynamic monomer/oligomer equilibrium in class A homodimers and heterodimers are of particular interest (Kroeger et al., 2003; Palczewski, 2010; Kleinau et al., 2016; Farran, 2017). In fact, according to their half-lives (determined by the association and dissociation rates) class A GPCR dimers are demonstrated to be often transient (Gurevich and Gurevich, 2008). This may contribute in explaining the opposing views regarding the role of class A GPCR monomers versus oligomers (Guidolin et al., 2018).

The amount of data proving the existence of GPCR heteromers showed a huge increase similarly to both development and diffusion of biophysical techniques able to detect the spatial proximity of protein molecules (Guidolin et al., 2015). They involve resonance energy transfer methods including bioluminescence and fluorescence resonance energy transfer (BRET and FRET, respectively), fluorescence complementation, total internal fluorescence microscopy, fluorescence correlation spectroscopy, assays based on bivalent ligands and proximity ligation assays (PLAs).

The basic molecular mechanism leading to the formation of the receptor assemblies are allosteric interactions (Kenakin et al., 2010; Fuxe et al., 2012a). As recently outlined by Changeux and Christopoulos (2016), the cooperativity that emerges in the action of orthosteric and allosteric ligands of the GPCR forming the complex provides the cell decoding apparatus with sophisticated dynamics in terms of recognition and signaling (George et al., 2002; Guidolin et al., 2018). It has been shown, for instance, that GPCR heterodimerization exerts effects by altering or fine-tuning ligand binding, signaling, as well as internalization of GPCR protomers (Bulenger et al., 2005; Satake et al., 2013; Gomes et al., 2016; Guidolin et al., 2018). Angiotensin II receptor (AT_1_) – bradykinin receptor (B_2_) heterodimer (detected in smooth muscle, omental vessel, and platelets) provides an example: angiotensin II triggers inositol triphosphate (IP3) accumulation in a manner that is much more potent and effective than in the cells showing the expression of AT_1_ alone; conversely, IP3 accumulation sustained by bradykinin is slightly weaker in cells expressing the AT_1_–B_2_ heterodimer with respect to the cells expressing only B_2_ (Satake et al., 2013).

Many evidences highlight that dimers may be formed by different mechanisms (Guidolin et al., 2018). Receptor complexes can arise in plasma membrane under the action of hydrophobic forces (Gahbauer and Böckmann, 2016). Receptor assembly may also occur in the endoplasmic reticulum (ER) before trafficking to the plasma membrane. ER is responsible of the structural quality of the synthetized receptors. Thus, only correctly folded receptors are allowed to exit while degradation occur for unfolded or misfolded proteins (Terrillon and Bouvier, 2004). Finally, recent data showed that GPCRs can be safely transferred by microvesicles from a source to a target cell where they become capable of recognizing and decoding their signal (Guescini et al., 2012).

Since GPCRs are implicated in several human diseases and represent major pharmacological targets, GPCR oligomerization process, determining variations in the processes of agonist recognition, signaling, and trafficking of participating receptors via allosteric mechanisms (Fuxe et al., 2012a), is of potential great importance for physiology and pharmacology, and new therapeutic strategies considering GPCR attitude to underwent toward several levels of receptor organization are under study (Farran, 2017).

Therefore, in a scenario where GPCR proteins are demonstrated to be present as monomer, the assumption that they may multimerize represents an interesting aspect to investigate. The purpose of the present article is to highlight indirect evidence of this with regard to the CB. In this tissue, indeed, the presence of multimeric GPCRs has not yet been consistently investigated. Thus, the main focus of the paper will be on possible homo-/heterodimers or mosaic receptors (already confirmed in other cell types) that could be present in the CB (type I and II cells, carotid sinus nerve afferents), with possible functional implications. The ‘working hypothesis’ of this paper could represent an interesting field of investigation aiming to clarify some aspects of chemoreception, neuromodulation and plasticity in the CB.

## Investigative Approaches to GPCR Complexes

As briefly discussed before, the term RRI emphasizes the occurrence of an interaction needing a direct physical contact between the involved receptor proteins responsible for the formation of multimeric assemblies of receptors (dimers or high-order oligomers) at the cell membrane level, acting as integrative input units of membrane-associated molecular circuits (Guidolin et al., 2018). Considerable efforts are currently being directed toward the identification of the most adequate strategy to discover such a supramolecular organization of GPCRs, but no single method is free from limitations giving rise to some discrepancy between various studies (Mackie, 2005; Lambert and Javitch, 2014). Thus, evidence obtained through multiple approaches with consistent results is needed to identify receptor complexes (Bouvier and Hébert, 2014).

In particular, an operational definition of RRI (Fuxe et al., 2010) can be exploited to devise experimental procedures fulfilling the following two measurable conditions:

1. The binding of a ligand to one receptor determines a detectable change in the biochemical features of another receptor, that includes ligand recognition, decoding, and trafficking processes;

2. Involved protomers have not only to be colocalized, but also in close proximity to each other (less than 10 nm), as indicated by specific techniques.

To explore the second point, biophysical and biochemical methods are presently available (see Kaczor and Selent, 2011 for a review).

### Biophysical Strategies

Biophysical methods may be divided into three macro-groups that are fluorescence/luminescence-based techniques, specialized microscopic techniques and X-ray crystallography.

As far as fluorescence/luminescence-based techniques are concerned, bioluminescence resonance energy transfer (BRET) and fluorescence resonance energy transfer (FRET) were among the earliest proximity-based assays of this type that were used in studying GPCR heteromerization (Fernández-Dueñas et al., 2012). Both techniques exploit the transfer of energy from a donor to an acceptor in a non-radiative manner (as a result of dipole–dipole coupling). If the donor is a fluorescent molecule with emission characteristics suitable to excite the acceptor, when they are in close proximity (∼10 nm) the result will be light emission from the acceptor fluorophore enabling the visualization of the subcellular location of specific receptor complexes.

Protein-fragment complementation assays have also been used to analyze GPCR heteromerization in living cells (Gandia et al., 2008). In this approach, cells are transfected with one GPCR fused to a fragment of fluorophore and another GPCR fused to a complementary fragment. Reconstitution of the active fluorophore would occur only if the two receptors were in close proximity. It is noteworthy that a combination of this technique with BRET or FRET can be devised to demonstrate the presence of GPCR trimers or tetramers (Gandia et al., 2008). In this respect, a specialized microscopic technique, namely atomic force microscopy, has also been explored to demonstrate the existence of GPCR complexes (Agnati et al., 2010).

However, all the mentioned techniques show limitations in answering questions concerning the dynamic nature of receptor complexes. Thus, more recently, the details of the spatial and temporal organization of GPCR complexes have been addressed by using specialized microscopic techniques allowing the direct observation of the state and behavior of individual proteins in living cells. They include total internal reflection fluorescence microscopy (Hern et al., 2010; Guidolin et al., 2018), fluorescence correlation spectroscopy (Chen et al., 2003; Guidolin et al., 2018) and photoactivated localization microscopy (PALM; Jonas et al., 2016).

X-ray crystallography represents a further significant experimental approach in the field. During the last years, crystallization techniques have been object of interest (Grisshammer, 2017), determining important consequences for the analysis of GPCR complexes as well as an increase of the number of experimentally assessed structures (Guidolin et al., 2018).

### Biochemical Strategies

Spatial and temporal co-expression of GPCRs in cells and tissues is a necessary condition for the formation of more complex systems. This aspect can be investigated following biochemical approaches. The abundance of a specific mRNA can be detected by Northern-blot analysis, *in situ* hybridization, RT-PCR or microarray analysis. Immunohistochemistry and co-localization analysis (Agnati et al., 2005) can be performed to detect GPCR co-expression and co-localization at protein level. The practical limit of this technique regards the necessity for high-quality antibodies also due to low GPCRs expression levels.

To identify possible complex formation, it is mandatory for biochemical studies extracting GPCRs from the membrane by use of detergent, although shielding their native conformation in detergent is not a trivial task (Park et al., 2004).

Radiation inactivation technique (or target size method) take advantage of gamma rays or high energy electrons to disrupt polypeptides in order to identify the molecular weight of singular components. It is based on the inverse relationship existing between the size of a macromolecule and its dose-dependent inactivation. Hence, the probability to destroy a molecule increases along with its mass: larger is a protein and lower is the energy required to destroy it (Rios et al., 2001). For what it concerns GPCRs, the main limitation of the method is that this variation may be related to interaction with other proteins instead of oligomerization.

To overcome this limitation, co-immunoprecipitation can be used (Skieterska et al., 2013). Interestingly, this method in combination with confocal microscopy and FRET was used to detect homooligomerization of the 5-HT_2C_ receptors as well as the 5-HT_4_ homodimerization in combination with BRET (Kaczor and Selent, 2011).

More recently, evidence of heteroreceptor complexes in native tissues has been provided by *in situ* PLA (Trifilieff et al., 2011). This technique could be adequate to highlight evidences regarding the presence of GPCR heteromers in tissue, giving new insights about basic biological mechanisms, heteroreceptor levels as well as their location. Thus, it allows to study the localization and modulation of heteroreceptor complexes, as formalin fixed tissue is used.

Cross-linking experiments are a further promising approach. They involve the use of assays based on bivalent ligands (agonists, antagonists, heteromer-selective antibodies, cross-linking reagents) allowing a direct targeting of the receptor complex (Yekkirala et al., 2013) in cells and tissues.

## Homo- or Hetero-Dimers Between A_1_ and/or A_2A_ Receptors

Adenosine receptor A_1_ homodimers have been identified in brain cortex of pigs by radioligand binding experiments (Ciruela et al., 1995). The capability of A_1_ receptors to form homodimers has also been demonstrated in Chinese hamster ovary (CHO) cells transfected with human A_1_ receptors (Gracia et al., 2008) and in transfected human embryonic kidney (HEK-293T) cells expressing similar levels of A_1_ receptor as the native tissue (Gracia et al., 2013). In bovine cortex, the existence of homomers was also shown through PLA (Gracia et al., 2013).

The effects of homodimerization on the receptor function can be inferred from ligand binding assays. A_1_ receptor activation inhibits adenylate cyclase and decreases cAMP concentration. Caffeine is a non-selective adenosine receptor antagonist. In case of low caffeine concentrations (when caffeine only binds to one protomer of the empty homodimer), it determines an increase of the agonist affinity for the other protomer in the A_1_ receptor homodimer; whereas, at high concentrations (when caffeine highly saturates both protomers of the homodimer) caffeine behaves like an A_1_ antagonist with a reduction of the agonist binding to the receptors. Thus, caffeine modulates A_1_ agonist-induced cAMP decrease in a biphasic manner. Interestingly, this is a particular behavior for a classical adenosine receptor antagonist like caffeine and is a pharmacological behavior explanation-lacking without taking into account receptor dimers as a minimal quaternary structure. Moreover, this pharmacological ability can also give explanations about the biphasic effects exerted at low and high concentration of caffeine on locomotor activity (Gracia et al., 2013).

As it regards the CB, adenosine is a well-known neurotransmitter produced in response to acute hypoxia. It can be directly released from type I cells, through an equilibrative nucleoside transporter, or it can be indirectly produced through breakdown of released ATP by ecto-5′-nucleotidase (Conde et al., 2012). Adenosine may act pre- and post-synaptically in the modulation of type I and afferents function. It enhances the firing pattern in the carotid sinus afferents through A_2A_ receptors (Conde et al., 2009a,b; Piskuric and Nurse, 2013). Some discrepancies between studies have been reported about A_1_ localization into the CB structures (Bairam et al., 2009). Rocher et al. (1999) described the presence of A_1_ in rabbit type I cells, through functional studies on cell cultures. Conversely, in the rat, post-synaptic localization has mainly been suggested as A_1_ receptors are significantly expressed in the cytoplasm of nodous and petrosal ganglia (Bairam and Carroll, 2005; Gauda et al., 2006) and immunostaining of whole CB did not permit to specifically localize immunoreaction in type I cells (Bae et al., 2005). Possible species-related differences have been suggested and further analyses also on human material would be necessary. However, to reassume, A_1_ homodimerization may be hypothesized in nerve fibers and probably in those species expressing A_1_ receptors in type I cells.

A_2A_ homodimerization has also been assessed in the plasma membrane of transfected HEK-293T cells by BRET, FRET, time-resolved BRET and immunoblotting and biotinylating experiments. Evidences highlight that more of 90% of A_2A_ receptors are present as homodimers. In intracellular areas, the presence of a certain amount of monomeric species has been highlighted, suggesting that that they assemble into dimers before reaching cell surface (Canals et al., 2004; Lukasiewicz et al., 2007).

In particular, Canals et al. (2004) demonstrated that A_2A_ homodimers are the functional species at the cell surface. Even though A_2A_ homodimerization is quite constitutive, it is further influenced by specific ligands: agonists (i.e., CGS 21680) and antagonists (i.e., SCH 58261; caffeine) increase and decrease it, respectively (Lukasiewicz et al., 2007).

Various authors confirmed the presence of A_2A_ receptor in the rat type I cells through several different techniques (RT-PCR studies, *in situ* hybridization analysis and immunohistochemistry) (e.g., Gauda, 2000; Kobayashi et al., 2000). Bairam et al. (2009) also demonstrated by Western-blot analysis the tendency of A_2A_ receptor to form homodimer in the CB similarly to the superior cervical ganglion and nucleus tractus solitarius. This is one of the few specific references about receptor dimerization in the CB.

However, there are not studies addressing the issue of how agonist/antagonists may modulate dimerization of adenosine receptors in the nerve fibers and/or type I cells or, conversely, how dimerization may play a role in their pharmacological actions. It must also be considered that environmental conditions (hypoxia, etc.) may modulate receptor expression as well as types of dimerization, potentially modulating pharmacological effects.

Apart from the above homodimers, the ability of A_1_ receptors to heterodimerize with A_2A_ receptors was also demonstrated in transfected cells by Ferré et al. (2008). Heterodimerization of these two protomers was assessed *in vitro* and *in vivo* by Ciruela et al. (2006), who confirmed the A_1_/A_2A_ heterodimer formation in rat striatal glutamatergic nerve terminals by immunogold detection and co-immunoprecipitation. Recently it has been shown that at the presynaptic membrane of cortico-thalamic glutamatergic terminals A_1_ receptors co-localizes and interacts with A_2A_ receptors, forming functional receptor heterodimer in the striatum (Fernández-Dueñas et al., 2017).

The eventual demonstration of A_1_/A_2A_ heterodimers in type I cells and/or nerve fibers would be of particular interest in terms of modulation of the glutamatergic transmission. In fact, heterodimerization between the adenosine receptors A_1_ and A_2A_, which are responsible for opposite signaling pathways (inhibitory and excitatory actions, respectively) (Stockwell et al., 2016), has been suggested to exert a function in fine-tuning modulation of striatal glutamatergic neurotransmission by adenosine. Since A_1_ receptor shows higher affinity for adenosine than A_2A_, but A_1_ agonist affinity decreases when A_2A_ is activated, the possibility exists that glutamate release may be inhibited or stimulated by a switch mechanism depending on low and high concentrations of adenosine, respectively (Ciruela et al., 2006; Doyle et al., 2012).

## Hetero-Dimers Between Adenosine (A_1_ and A_2A_) and Purinergic (P2Y_1_, P2Y_2_, P2Y_12_) Receptors

P2Y_1_ (Xu et al., 2005; Tse et al., 2012) and P2Y_12_ (Carroll et al., 2012) receptors have been demonstrated in type I cells, where they inhibit the hypoxia-induced rise in intracellular Ca^2+^. Conversely, in type II cells, P2Y_2_ receptors have been demonstrated (Xu et al., 2003), which produces a rise in intracellular Ca^2+^.

Purinergic receptors have also been reported to form homodimers and hetero-dimers with other purinergic and adenosine receptors (e.g., Schicker et al., 2009). As previously detailed, A_2A_ receptors are expressed in type I cells (Gauda, 2000; Kobayashi et al., 2000) and A1 receptors have been reported in the type I cells of some species (rabbit) (Rocher et al., 1999), although not confirmed in others (rat) (Gauda et al., 2000, 2006). Thus, in type I cells, many RRIs can be hypothesized: P2Y_1_/P2Y_1_, P2Y_1_/P2Y_12_, P2Y_12_/P2Y_12_, P2Y_1_/A_1_, P2Y_1_/A_2A_, P2Y_12_/A_1_, P2Y_12_/A_2A_ (e.g., Yoshioka et al., 2001; Nakata et al., 2005, 2010; Schicker et al., 2009). Physical interactions between A_1_ and P2Y_1_ receptors, for instance, produce a conformational change in the A_1_ binding pocket with acquisition of P2Y_1_-like agonistic pharmacology, i.e., a P2Y_1_ agonist may bind to the A_1_ receptor and produce an inhibition of adenylate cyclase which is prevented by A_1_ antagonist (Fuxe et al., 2008). All the above RRIs would be particularly intriguing as they would represent other ways of reciprocal modulation between purinergic and adenosine neurotransmission both in type I and II cells.

## Homo- and Hetero-Dimers Between Dopamine Receptors (D_1_ and D_2_)

According to co-immunoprecipitation data gathered from both rat brain and cells showing co-expression of the D_1_ and D_2_ receptors, occurs the idea they may constitute the same heteromeric protein complex; moreover, according to immunohistochemistry studies, these receptors are convincingly co-expressed and co-localized within neurons of human and rat brain (Lee et al., 2004). For instance, a significant neuronal subpopulation in rat nucleus accumbens co-expresses D_1_ and D_2_ receptors, which can form a D_1_/D_2_ receptor complex (Hopf et al., 2003; Hasbi et al., 2018). Rashid et al. (2007) also provided evidences concerning their co-expression in human embryonic kidney cells. In parallel, Rashid et al. (2007) and Hasbi et al. (2018) demonstrated the ability of D_1_ and D_2_ receptors to oligomerize *in vivo* in rodent and monkey striata, respectively, through *in situ* PLA, *in situ* FRET and co-immunoprecipitation.

D_1_ and D_2_ monomers are coupled to G_s_ and G_i_ proteins, respectively, and they are usually considered to exert opposite effects at the cellular level (Hopf et al., 2003). Conversely, the heterodimer D_1_/D_2_ is coupled to G_q/11._ In the nucleus accumbens, the activation of this heterodimer-specific pathway determines an increase of calcium/calmodulin-dependent protein kinase IIα in contrast with the effect produced by the activation of the Gs-D_1_ receptor (Rashid et al., 2007).

Numerous studies documented the presence of D_1_ receptor in the CB of various animals through Northern blot analysis, RT-PCR and pharmacological studies, although the precise location has not been verified (Almaraz et al., 1991; Bairam et al., 1998, 2003; Schlenker, 2008). Almaraz et al. (1991) suggested expression in blood vessels. Bairam et al. (1998) suggested that D_1_ receptor could be expressed in nerve terminals (sensitive and sympathetic). Conversely, D_1_ receptors were identified in hamster type I cells through immunohistochemistry (Schlenker and Schultz, 2011). D_2_ has been demonstrated in type I cells and nerve fibers of the rat CB by double immunofluorescence (Wakai et al., 2015) and its expression is higher than D_1_ (Bairam et al., 1998). The contemporary presence of D_1_ and D_2_ in type I cells and nerve fibers would support the hypothesis of heterodimerization in these structures. Exogenous dopamine may also inhibit Ca^2+^ responses in type II cells, supporting the presence of corresponding receptors (Leonard and Nurse, 2017; Leonard et al., 2018) and of possible dimerization.

Huey and Powell (2000) explored the consequences of chronic hypoxia on D_2_ receptor expression in the arterial chemoreflex pathway. As regards the CB, the authors observed that the regulation of D_2_-receptor mRNA expression is time-dependent. Briefly, an initial increase of D_2_ receptor mRNA levels was observed after 6 and 12 h, with a subsequent decrease (24, 48 h) and a final significant increase after 168 h of hypoxia. In a noteworthy and analog study, Bairam et al. (2003) considered the effects induced by hypoxia on the expression levels of both D_1_ and D_2_ receptor mRNAs. The authors highlighted that in 1-day-old rabbits hypoxia affects the expression of D_2_- or D_1_-receptors mRNA in a manner that is age-dependent. D_2_- and D_1_-receptors transcript levels increased and decreased after exposure to moderate (15% O_2_) and severe (8% O_2_) hypoxia, respectively. Conversely, D_2_- and D_1_-transcript levels decreased independently of hypoxia intensities in adult rabbits. Unlike D_1_ receptors, changes in D_2_ receptor mRNA levels were independent on exposure time. The D_2_ role in the CB has been suggested not to be key under normal conditions, but rather in situations of chronic hypoxia, where D_1_ and D_2_ receptors density tends to change as previously discussed (Bairam et al., 2003).

The above changes in the expression of the different dopamine receptors after hypoxia suggest the possibility of consequent changes in the amount of D_1_/D_2_ heterodimers. It is intriguing the idea that D_1_/D_2_ heterodimer formation may be under the influence of environmental stimuli, mainly hypoxia but possibly others, in the CB.

## Hetero-Dimers Between Adenosine (A_1_ and A_2A_) and Dopamine (D_1_ and D_2_) Receptors

The co-localization of A_1_ and D_2_ receptors has been showed by immunofluorescence in rat cerebral cortex neurons (Gines et al., 2000). Moreover, the presence of A_1_/D_1_ complexes was detected in mouse fibroblast Ltk-cells, transfected with human A_1_, D_1_, and D_2_. While the formation of A_1_/D_1_ heteromers was confirmed by coimmunoprecipitation, A_1_ did not seem to form heterodimers with D_2_. A_1_ and D_1_ receptors, separately, also tend to form homodimers; however, the preferred form of dimerization for these two receptors still needs to be elucidated (Agnati et al., 2003). Additionally, A_1_/D_1_ dimerization was also confirmed by FRET in HEK-293T cells (Shen et al., 2013).

Due to the possible presence of both A_1_ and D_1_ receptors in type I cells, a role by A_1_/D_1_ dimerization may be also be supposed. In particular, A_1_/D_1_ dimerization could represent a way of integration between agonists and/or antagonists for the two different monomers. For instance, Gines et al. (2000) suggested that A_1_/D_1_ heteromerization may have a role in the antagonistic modulation of D_1_ receptor in the brain, thus having an impact on desensitization mechanism of D_1_ and receptor trafficking.

A_2A_/D_2_ heterodimers have also been demonstrated both in transfected SH-SY5Y (Hillion et al., 2002; Xie et al., 2010) and HEK-293T cells (Navarro et al., 2014), by immunoprecipitation followed by Western-blotting (SH-SY5Y) and BRET (HEK-293T). In addition, A_2A_/D_2_ heterodimers were identified in neuronal primary cultures of rat striatum by PLA (Navarro et al., 2014) and cAMP accumulation experiments (Hillion et al., 2002). A_2A_/D_2_ heterodimers have also been demonstrated in the mammalian striatum, with particular reference to the striatal enkephalin-containing GABAergic neurons that project to the globus pallidus and comprise the so-called indirect pathway (Fink et al., 1992; Fuxe et al., 1998; Schiffmann et al., 2003; Trifilieff et al., 2011; Doyle et al., 2012; Atack et al., 2014). Thus, A_2A_/D_2_ heterodimers play an important role in the modulation of GABAergic striato-pallidal neuronal function (Bonaventura et al., 2015). In particular, antagonistic A_2A_-D_2_ RRIs occur in the heterodimer, as demonstrated in striatal membrane preparations after incubation with the A_2A_ agonist CGS21680, leading to a decrease of the affinity of the high affinity D_2_ agonist-binding site (Fuxe et al., 1998; Guidolin et al., 2018). Relationships between A_2A_ and D_2_ and adenosine/dopamine cross-talks have been proposed as possible new therapeutic approaches for Parkinson’s disease, schizophrenia, and drug addiction (Canals et al., 2003; Guidolin et al., 2015).

As previously discussed, both A_2A_ and D_2_ are expressed in type I cells of the CB, supporting the hypothesis of A_2A_/D_2_ heterodimer formation also in these cells. The reciprocal influences of the two receptor monomers in the A_2A_/D_2_ complex would be particularly intriguing, as adenosine and dopamine are among the main excitatory and inhibitory neurotransmitters in the CB. Some authors have proposed a functional interaction between A_2B_ and D_2_ receptors. In particular, if adenosine levels would become too high, activation of the low affinity A_2B_ would lead to increased dopamine secretion from type I cells (Conde et al., 2006; Livermore and Nurse, 2013) and inhibition of sensory discharge through pre- and post-synaptic D_2_ receptors (Conde et al., 2009a,b, 2012; Zhang et al., 2017; Nurse et al., 2018). RRI between A_2A_ and D_2_ could represent an opposite way of neuromodulation, increasing signaling efficiency in the presence of low adenosine levels, through allosteric receptor–receptor inhibition of D_2_ signaling.

In rat type I cells, Conde et al. (2008, 2009b) also proposed a A_2B_-D_2_ receptor interactions on the basis of *in vitro* pharmacological experiments, although they did not demonstrate if physical RRI occur but only demonstrated an interaction at the adenylyl cyclase level. In particular, they showed that D_2_ agonists decrease catecholamine release and inhibit cAMP production in the CB and that these effects are prevented by A_2B_ agonists; conversely, D_2_ antagonists increase the release of catecholamines, this phenomenon being also prevented by A_2B_ antagonists. A_2B_-D_2_ RRI, however, have not yet been demonstrated to date in any cell type.

## Homo- and Hetero-Dimers Between Opioid Receptors (μOR and δOR)

μOR forms homodimers (Lopez and Salome, 2009) with an elevated stability, as demonstrated in transfected HEK-293 cells by BRET and radioligand binding assays (Wang et al., 2005). μOR/μOR homodimerization occurs prior of transportation to the cell membrane. Sarkar et al. (2012) also proved μOR homodimerization in rat NK cells through Western-blot analysis and immunoprecipitation as well as immunohistochemistry and immunofluorescence.

Also δOR homodimerization was confirmed by Western-blotting and co-immunoprecipitation in different transfected cellular systems, such as CHO cells and COS cells (Cvejic and Devi, 1997). Data obtained by cross-linking experiments in CHO cells indicated that homodimerization does not depend on the expression level of the receptor. Subsequently, the occurrence of homodimers was also assessed in HEK-293T cells by BRET (Johnston et al., 2011) and in rat NK cells by Western-blot assays, immunoprecipitation, immunohistochemistry, and immunofluorescence (Sarkar et al., 2012).

δOR/μOR heterodimers have also been demonstrated (George et al., 2000; Gomes et al., 2002; Sarkar et al., 2012). The heteroreceptor complexes exhibit distinct pharmacological properties from that of the monomers. Low non-signaling doses of δ- or μ-ligands potentiate the binding and signaling of the receptors and the affinity of endomorphin-1 and δ-selective agonists is increased in the heteroreceptor complex, when compared to monomers (Kabli et al., 2010). These properties appeared linked to changes in the signaling pathways activated by the heteroreceptor complexes as compared to individual receptors (Gomes et al., 2013). In NK cells, in which δOR and μOR proteins exist as homo- and heterodimers, differences in cell function were observed depending on the prevalent type of receptor dimerization (Sarkar et al., 2012).

As suggested by Cvejic and Devi (1997) the dimerization also plays an important role in the internalization of the δOR receptor which in its monomeric form may not be internalized. This could be related to the mechanism that takes place after long-term exposure to opioid ligands. Dimerization of δOR receptors seems to be agonist dependent.

As it regards the CB, both μOR and δOR are expressed in type I cells (Ichikawa et al., 2005; Ricker et al., 2015). The expression of both receptors in type I cells suggests the occurrence of homo- and hetero-dimerization, with possible pharmacological implications. Perivascular nerve fibers have also been reported to express δOR (Ichikawa et al., 2005) although further studies will be necessary for detection of other opioid receptor types.

## Hetero-Dimers Between Dopamine (D_1_) and Opioid (μOR) Receptors

Co-immunoprecipitation, BRET and cross-antagonism assays demonstrated the existence of D_1_/μOR heterodimers in both transfected HEK-293 cells and ventral striatum (Tao et al., 2017). D_1_ receptor antagonist can antagonize μOR-mediated signaling and function in a dopamine-independent manner, mostly likely via allosteric interactions through the D_1_/μOR heteromer. D_1_ antagonist (SCH23390) prevented opiate-induced activation of G-protein, inhibition of adenylyl cyclase, phosphorylation of ERK 1/2 and expression of c-fos in transfected cells expressing both receptors and in striatal tissues from wild type, but not DR KO, mice. The ability of an antagonist to inhibit signals originated by stimulation of the partner receptor is a biochemical characteristic that has been described for receptor heterodimers (cross-antagonism) (Tao et al., 2017).

As discussed above, D_1_ receptor and μOR were both assessed in the CB, supporting the hypothesis of corresponding RRI. Moreover, changes in the dynamic monomer/oligomer equilibrium may be postulated on the basis of environmental stimuli, as D_1_ expression levels, for instance, decrease in hypoxia conditions and along with exposure time (Bairam et al., 2003).

## Hetero-Dimers Between Dopamine (D_1_ and D_2_) and Histamine (H_3_) Receptors

Koerner et al. (2004) assessed the presence of histamine receptors (H_1_, H_2_, H_3_) in the CB through RT-PCR studies. In particular, H_3_ has been detected in rat type I cells by immunohistochemistry (Lazarov et al., 2009; Del Rio et al., 2009; Thompson et al., 2010). H_3_ antagonism results in increased chemosensory activity (Del Rio et al., 2009) and H3 activation inhibits the intracellular Ca^2+^ signaling mediated by activation of muscarinic receptors in type I cells (Thompson et al., 2010). On the basis of the above and following findings, histamine receptors could also be included in the series of GPCRs with potential oligomerization in the CB.

In fact, Ferrada et al. (2009) demonstrated the D_1_–H_3_ receptor heteromerization in mammalian transfected cells (HEK-293) by BRET and binding assays as well as in the neuronal cell model SK-N-MC by radioligand experiments. The presence of this heteromer in the brain (striatum) was then also proved by Moreno et al. (2011).

An antagonist acting on one of the receptor units that constitute the D_1_–H_3_ receptor heteromer can cause changes in conformation of the other receptor unit, thus, blocking specific signals that originate in the heteromer. This mechanism could be responsible for unsuspected GPCR antagonists-related therapeutic potentials (Ferrada et al., 2009). D_1_/H_3_ receptor heteromers work as processors integrating dopamine- as well as histamine-related signals involved in controlling the function of striatal neurons of the direct striatal pathway (Moreno et al., 2011).

A strong and selective heteromeric interaction between D_2_ and H_3_ was also assessed by radioligand binding experiments in striatal membrane preparations and in co-transfected HEK-293 cells by BRET (Ferrada et al., 2008). According to agonist/antagonist competition experiments, a significant decrease of D_2_ receptors affinity for the agonist was encountered after stimulation of H_3_ receptors with several H_3_ agonists (Ferrada et al., 2008).

## Hetero-Dimers Between Dopamine (D_2_) and Serotonin (5-HT_2A_) Receptors

5-HT_2A_/D_2_ heteroreceptor complexes were demonstrated by PLA and immunofluorescence in co-transfected HEK-293T cells (Lukasiewicz et al., 2010; Borroto-Escuela et al., 2014), as well as in discrete regions of the ventral and dorsal striatum and nucleus accumbens (Borroto-Escuela et al., 2014), medial prefrontal cortex as well as in the pars reticulate of the substantia nigra in rat (Lukasiewicz et al., 2010).

The functional properties of this complex are not yet completely clear (Lukasiewicz et al., 2010). However, ligands with high 5-HT_2A_/D_2_ selectivity and partial agonistic activity on D_2_ have been recently identified as new antipsychotic drugs which do not significantly induce extrapyramidal side effects (Möller et al., 2015).

Serotonin is released by type I cells and it acts in a autocrine/paracrine manner on 5-HT_2_ receptors which are localized in type I cells, as proved by immunofluorescence and pharmacological/functional studies in the rat CB (Zhang et al., 2003; Jacono et al., 2005; Liu et al., 2011; Yokoyama et al., 2015). Possible expression of 5-HT_2A_ in carotid sinus nerve terminals has also been reported (Nurse et al., 2018). Serotonin is also considered to be involved in long-term facilitation of CB sensory discharge due to chronic intermittent hypoxia (Peng et al., 2009; Prabhakar, 2011). The presence of 5-HT_2A_ and D_2_ in type I cells suggests the possibility of heterodimer formation, with functional roles to be investigated. 5-HT_2A_ and D_2_ mediate excitatory and inhibitory effects on type I cells and the role of dimerization in the modulation of the two response types is particularly intriguing.

## Hetero-Dimers Between Dopamine (D_2_) and Neurotensin (NTS_1_) Receptors

Among CB receptors possibly interacting with D_2_ receptor there is also the neurotensin receptor 1 (NTS_1_), which has been demonstrated by immunohistochemistry in rat and human type I cells (Porzionato et al., 2009). D_2_-NTS_1_ complexes have also been highlighted in living HEK293T cells by BRET technology (Borroto-Escuela et al., 2013b). Through these RRI neurotensin reduces dopamine binding and function at D_2_ receptor, moving dopamine transmission toward D_1_ receptor, which is not antagonized by NTS_1_ (Borroto-Escuela and Fuxe, 2017).

## Homo- and Hetero-Dimers Between Melatonin Receptors (MT_1_ and MT_2_)

MT_2_ receptors have the ability to form homodimers, as first demonstrated in HEK-293T transfected cells through BRET approaches (including single-cell BRET experiments), as well as Western-blotting and co-immunoprecipitation assays (Ayoub et al., 2002). However, it has also been observed that MT_2_ receptors tend to preferentially form heterodimers with MT_1_, as proved by BRET techniques (BRET donor saturation assay) in the same cell model (Ayoub et al., 2004).

The homodimerization of MT_2_ receptors is not modulated by ligands as it can exist in a constitutive manner in living cells. However, the speculated role of MT_2_ dimers is that they are required for explicating biological functions of cells being considered functional signaling units. Modulation of the signaling and trafficking occurs similarly to the GABA_B_ receptors (Ayoub et al., 2002).

As regards the presence of MT receptors in the CB, an *in situ* hybridization study showed MT_2_ expression in the rat type I cells whereas there are not yet data about MT_1_ (Tjong et al., 2004). Thus, to date, we can suppose MT_2_ homodimerization in type I cells although further analyses could be of interest for MT_1_ detection.

## Galanin Receptor (Gal_1_, Gal_2_) Heteromers

Galanin (Mazzatenta et al., 2014) and galanin receptors 1 (Gal_1_) and 2 (Gal_2_), but not 3 (Gal_3_), (Porzionato et al., 2010) have been demonstrated by immunohistochemistry in rat type I cells. Galanin is known to regulate the differentiation of neural stem cells and plasticity responses in the nervous system (e.g., Cordeo-Llana et al., 2014) and it has been suggested that galanin expression in chemoreceptor cells could provide a signal for neurogenesis and chemoreceptor cell differentiation (Di Giulio et al., 2015; Mazzatenta et al., 2016).

As it concerns this paper, galanin receptors are particularly intriguing because evidence is given about the possibility of Gal_1_-Gal_2_, D1-Gal_1_, Gal-NPY_*Y*1_, Gal_1_-Gal_2_-NPY_*Y*2_ or Gal_1_-Gal_2_-AT_1_ heteromers, which can be postulated in type I cells (reviewed in Fuxe et al., 2012b).

Although there are not specific data about the expression of the different NPY receptor types in the CB, NPY immunoreactivity has been identified in nerve fibers as well as type I cells of dog, monkey and rat CB (Oomori et al., 1991, 2002). In the rat CB, a reduction in NPY-immunoreactive type I cells was observed from postnatal week 2 onward; conversely, NPY-immunoreactive fibers mainly increase since week 2 after birth (Oomori et al., 2002).

The local renin-angiotensin system in the CB has been extensively studied in the past years (e.g., Fung et al., 2002; Fung, 2014; Lam et al., 2014) and angiotensin II receptor type 1 (AT_1_) has been identified in rat type I (Fung et al., 2001; Atanasova et al., 2018) and type II (Murali et al., 2014) cells. In the rat, AT_1_ receptors are also up-regulated in response to hypoxia (Fung et al., 2002).

## Homo- and Hetero-Dimers Between Endothelin Receptors (ET*_A_* and ET*_B_*)

Endothelin 1 (ET-1) receptors type A (ET*_A_*) and B (ET*_B_*) may form homo- and heterodimers, although the functional implications of different dimerization are still largely unknown. Their behavior was studied after expression in transfected HEK-293T cells by immunoprecipitation, immunoblotting and FRET (Evans and Walker, 2008). These results confirmed the previous data by Gregan et al. (2004) who adopted FRET too, co-localizing ET-1 receptors at the plasma membrane. Furthermore, the latter speculated that ET-1-receptors homodimerization (as well as homo-oligomerization) is constitutive and ligand-independent (Evans and Walker, 2008).

Type I cells synthetize and release ET-1, which may act in autocrine/paracrine manner on the same type I cells through both the above receptors. Chronic continuous hypoxia upregulates ET-1 and ET*_A_* receptor, suggesting a critical role in chronic hypoxia-induced increased chemosensitivity in the rat CB (Chen et al., 2002). Chronic intermittent hypoxia is also known to enhance the CB chemosensory and ventilatory responses to acute hypoxia and produce long-term sensory potentiation of chemosensory discharges (e.g., Peng et al., 2003, 2013; Rey et al., 2004; Pawar et al., 2009). Chronic intermittent hypoxia has been reported to determine an upregulation of ET-1 and ET*_B_* receptor, but not of ET*_A_* receptor, in adult cats (Rey et al., 2007); conversely, upregulation of ET-1 and ET*_A_* receptor, but not ET*_B_* receptor, have been reported in neonatal (Pawar et al., 2009) and adult (Peng et al., 2013) rats.

Endothelin rise intracellular Ca^2+^ in cultured type II cells (Murali et al., 2015). Stimulation of ET*_B_* receptors by ET-1 has been reported to play a role in proliferation of stem cells derived from type II cells and CB hyperplasia following chronic hypoxia (Platero-Luengo et al., 2014).

Thus, RRIs between ET-1 receptors can be hypothesized in type I and II cells. Hypoxia-induced changes in the expression of two different receptor types, together with the involvement of ET-1 in functional/structural modifications, suggests the idea that changes in the monomer/(homo/hetero)-dimer equilibrium may also play a role.

ET*_B_* receptor has also been reported to undergo physical interactions with AT_1_ receptor located in the cells of renal proximal tubule (Zeng et al., 2005). RRI between the two receptors would be particularly intriguing in the CB, due to their roles in hypoxia responses.

## Hetero-Dimers Between GABA (GABA_*B*2_) and Muscarinic (M_2_) Receptors

Boyer et al. (2009) demonstrated by FRET that GABA_*B*2_ is co-localized and directly associates with M_2_ receptor in neuronal PC12 cells. In parallel, the authors observed that in another cell model, i.e., HEK-293T, GABA_B1_ is also required for GABA_*B*2_/M_2_ heterodimerization, contrary to what was observed in PC12. Moreover, through co-immunoprecipitation and immunostaining experiments, the authors supported that signaling complexes GABA_*B*2_/M_2_ exist also *in vivo* in the brain cortex. The association seems to be specific since GABA_*B*2_ did not associate closely with other related muscarinic receptors (M_1_) or with a different GPCR (μOR).

The findings that M_2_ and GABA_*B*2_ are co-localized and associate in cortical neurons, which overlap with brain regions that receive cholinergic projections, suggest that the heterodimer is involved in a novel mechanism for enhancing cholinergic signaling in the brain. In fact, expression of GABA_*B*2_ in M_2_-expressing neurons would allow some neurons to maintain muscarinic signaling during elevated or chronic agonist exposure (Boyer et al., 2009).

Type I cells of the CB express both GABA_B1_ and GABA_*B*2_ subunits, as confirmed by RT-PCR studies. In addition to molecular biology evidences, localization of GABA_*B*2_ receptor subunits in sections of the CB was also assayed by immunofluorescence which assessed positive immunoreactivity for the receptor subunit in type I clusters (Fearon et al., 2003). Shirahata et al. (2004) demonstrated the expression of M_2_ mRNA and the presence of the receptor protein, by RT-PCR and immunohistochemistry, respectively. In particular, regarding localization, immunohistochemical analysis highlighted M_2_ presence in type I cells and petrosal afferent terminals. GABA and acetylcholine (Dasso et al., 1997) show inhibitory and excitatory actions, respectively, on type I cells. The possible occurrence of dimerization may have a role in the reciprocal modulation of actions of the two neurotransmitters.

Muscarinic receptors are also present in type II cells, as muscarinic agonists elicit an increase of intracellular Ca^2+^ levels in these cells (Tse et al., 2012; Murali et al., 2015), but there are not data about the possible expression of GABA receptors too.

## Cannabinoid Receptor (CB1) Heteromers

The CB_1_ receptor was found to be expressed in the CB by techniques such as RT-PCR, immunohistochemistry and *in situ* hybridization, although its levels were relatively low (McLemore, 2004). These findings suggest that endocannabinoids may have an impact on blood flow regulation in the CB, therefore affecting the oxygen pressure and respiratory control. However, the CB_1_ function in CB needs to be further investigated, as data present in another study on CB_1_ were somewhat discording (Roy et al., 2012).

The CB_1_ can form heterodimers with many other different GPCRs which are also assessed to be present in the CB; among these δOR, μOR, A_2A_ and D_2_ receptors. In particular, the ability of CB_1_ and D_2_ receptors to form heterodimers was demonstrated in co-transfected cells (HEK-293T) through co-immunoprecipitation and FRET techniques (Kearn, 2005; Marcellino et al., 2008). It is noteworthy that the activation of CB_1_-D_2_ receptor heterodimer can have completely opposite effects than activation of the individual receptors.

The above receptors have also been demonstrated to oligomerize in receptor mosaics so that the presence of these complexes may also be postulated for the CB. In particular, the existence of a CB_1_-D_2_-A_2A_ receptor mosaic has been demonstrated, where CB_1_ receptor activation removes the D_2_ inhibition of the A_2A_ receptor signaling (Fuxe et al., 2008; Marcellino et al., 2008). The above receptor mosaicism could represent a further way of subtle reciprocal modulation between different neurotransmitters.

## Heterocomplexes Between GPCR and Other Receptor Types

G protein-coupled receptors may undergo direct interactions with other membrane receptors, such as ion channel receptors or receptor tyrosine kinases (Borroto-Escuela et al., 2016). Some of these complexes can be postulated in the CB, with possible roles in modulation of neurotransmission and plasticity.

For instance, NMDA receptor subunits 1, 2A and 2B have been detected in CB, through RT-PCR, and type I cells, through immunohistochemistry. Chronic intermittent hypoxia also increases the expression of NMDA_1_ and NMDA_2B_ receptors (Liu et al., 2009). It is noteworthy that D_1_ receptors can regulate the function of the NMDA receptor by means of direct protein–protein interactions between the carboxyl terminals of D_1_ receptor and NMDA_1_ and NMDA_2A_ (Lee et al., 2002; Li et al., 2010), stimulating NMDA receptor-mediated long-term potentiation (Nai et al., 2010).

FGF receptor 1, which is a tyrosine kinases receptor, has been reported to undergo direct RRI with A_2A_ receptor (Flajolet et al., 2008; Borroto-Escuela et al., 2013a). In particular, as demonstrated in PC12 cells (showing a series of similar properties with type I cells), contemporary activation of the two receptors, but not the single ones, induces activation of the MAPK/ERK pathway, differentiation and neurite extension (Flajolet et al., 2008). The majority of human CB’s type I cells have shown a weak to moderate cytoplasmic immunostaining for FGF_1_ receptor (Douwes Dekker et al., 2007). In particular, FGF_1_ receptor immunoreactivity was shown in type I cells in postnatal rat CB cultures, in both normoxic and hypoxic conditions, as well as in bFGF presence or absence (Paciga and Nurse, 2001). bFGF increased both inward Na^+^ and outward K^+^ currents after 2 days of treatment on cultured type I cells from E18-19 rat pups (Zhong and Nurse, 1995). In cultures from rat E17-E19 CB, bFGF increases survival and BrdU incorporation; in postnatal P1-P3 cultures, bFGF still stimulates DNA synthesis but does not affect survival. In fetal rat glomus cells, bFGF stimulates neuronal differentiation, producing neurite outgrowth and inducing neurofilament immunoreactivity; these changes can no longer be detected in postnatal cultures (Nurse and Vollmer, 1997). Given the concomitant expression of A_2A_ receptors in type I cells, an heterocomplex could be hypothesized. Moreover, the above developmental changes in the FGF action could partially derive by changes in the monomer–heteromer equilibrium. It is known, for instance, that the expression of A_2A_ receptors in the rat type I cells decreases by PN14 (Gauda et al., 2000).

It is noteworthy that A_2A_-D_2_-FGF receptor mosaic has also been highlighted in other cell types (Fuxe et al., 2010) and could represent a further way of integration between neuromodulation and plasticity mechanisms.

## Conclusion and Future Perspectives

The CB is characterized by the production and release of many different neurotransmitters and neuromodulators which act on various receptor types identifiable on type I and II cells and nerve fibers. Although the main receptors involved in conveying excitatory stimuli from type I cells to afferent nerve fibers are ionotropic (nicotinic, P2X_2_/P2X_3_), a wide series of GPCRs is also expressed in the various structures involved in chemoreception, exerting modulation of neurotransmission. In other transfected and native cell types, experimental evidence has been provided about the existence of RRIs between GPCRs, with production of homodimers, heterodimers or high-order complexes (receptor mosaics) which may modify ligand binding, signaling and internalization of the protomers. In the present paper, we have reviewed most literature data reporting the contemporary expression in type I cells, type II cells or afferents of GPCRs which are able to physically interact with each other to form homo/hetero-complexes. This is a prerequisite for postulating dimerization and/or oligomerization in the CB. RRIs are particularly intriguing to be hypothesized in the CB, where *in vitro*/*in vivo* pharmacological/functional data are available about cross-talks between different neurotransmitters/neuromodulators or ‘paradoxical’ response changes with different concentrations/doses. At least in some cases, such findings could be interpreted through the existence of direct RRIs. Nevertheless, few authors have considered the possibility of di/oligo-merization in the CB (Conde et al., 2008, 2009b) and to the best of our knowledge there are not studies addressing this aspect through up-to-date methodology (see corresponding paragraph). The literature data reviewed in the present paper support the possibility of RRI in the CB and stress the potential implications of di/oligomerization for chemoreception, neuromodulation and plasticity. It is known, in fact, that the expression of certain receptors varies in response to development or environmental stimuli (hypoxia, hyperoxia, etc.); changes in the dynamic monomer/oligomer equilibrium may be the consequence, with correlated functional implications.

In the present paper, we have collected indirect evidence of our ‘working hypothesis,’ identifying the most significant homo/hetero-complexes which would be worthwhile to be studied in the CB. The direct identification of homo/hetero-complexes in type I, type II and afferent terminations and the characterization of their functional role and relevance in the chemoreception could represent an exciting field of investigation. As previously stated, demonstration of RRIs in the CB should include (1) assessment of colocalization in “close proximity” of two or more receptors and (2) detectable biochemical/functional change in one receptor induced by the binding of a ligand to another receptor. We have here revised literature data highlighting colocalization of different receptors in the various elements of the CB (i.e., type I and II cells, nerve terminals). In addition, colocalization of other receptors could be preliminarily investigated through double immunofluorescence or *in situ* hybridization, both in CB tissue samples and cell cultures.

However, colocalization is just a necessary condition to have direct RRIs and the demonstration of close proximity (i.e., a distance lower than 10 nm) between receptor molecules must be provided. To date, many methods of both biochemical and biophysical nature are available to accomplish this task and most of them could be applied to CB cell cultures. In particular, BRET, FRET or atomic force microscopy could be considered the most useful approaches. Interestingly, *in vitro* models allow to detect changes in cultured CB cells as a response to external pharmacological or environmental (hypoxia, hyperoxia, etc.) stimuli; we have hypothesized that RRIs may increase or decrease in response to external actions. Dynamic modifications of RRIs could also be analyzed in living cells through experimental techniques such as total internal reflection fluorescence microscopy, fluorescence correlation spectroscopy and PALM. Apart from cell cultures, heterocomplexes could be identified in native formalin-fixed CBs through *in situ* PLA, an approach that would even permit the identification of eventual changes in RRIs with respect to experimental conditions of *in vivo* models.

Once demonstrated physical RRIs, functional approaches in cell cultures would have to verify how the agonist/antagonist to one receptor may modify the response of the other receptor(s) toward the corresponding agonists/antagonists.

In conclusion, many *in vitro* and *in vivo* models are available, for research in CB structure/function, which represent adequate material for the application of consistent methods of analysis of potential RRIs.

## Author Contributions

All the authors contributed to the revision of the literature and discussion of the hypothesis proposed. All the authors read and approved the final version of the manuscript.

## Conflict of Interest Statement

The authors declare that the research was conducted in the absence of any commercial or financial relationships that could be construed as a potential conflict of interest.
